# Supplementary material for: The Genetic Basis of Obesity and Related Metabolic Diseases in Humans and Companion Animals
Source: Genes (Basel). 2020 Nov 20;11(11):1378. doi: 10.3390/genes11111378 (PMC7699880; doi:10.3390/genes11111378)
Supplement: Supplementary file 1 [file genes-11-01378-s001.pdf]

Table S1 - Candidate genes and gene variants for obesity and related metabolic disease in companion animals. Gene variants used to test for association with obesity/obesity-related traits in candidate gene studies in companion animal species. N/A = not available, - = not applicable. *POMC*, proopiomelanocortin gene; *FTO*, FTO alpha-ketoglutarate dependent dioxygenase gene; *INSIG2*, Insulin induced gene 2; *FFAR4*, Free fatty acid receptor 4 gene; *GPR120*, G-protein coupled receptor 120 gene; *MC4R*, Melanocortin 4 receptor gene; *MC3R*, Melanocortin 3 receptor gene; *PPARG*, Peroxisome proliferator activated receptor gamma gene; *TNF*, Tumor necrosis factor gene; *IL6*, Interleukin 6; *RETN*, Resistin gene; *FAM174A*, Family with sequence similarity 174 member A gene; BCS, body condition score.

Gene	Species: Breed(s)	Genome build used	Variant(s) referenced in original paper	Alternative variant name used in current review (realigned to CanFam 3.1 where applicable)	Variant consequence	Trait/association tested	Reference
POMC	*Canis familiaris:* Labrador retriever, flat-coated retriever	CanFam 3.1	17:19431664, G>T, S239I	c.716C>A, p.Ser239Ile, rs852254866	Nonsynonymous, missense	No significant association found with BCS	Raffan et al., 2016 [1]
			17:19431807, GCGCCGGCCCGGGA>- , P187fs, (17:19431807-19431821)	c.560_573del AGGGCCCCGCCGCG, p.Q186fs, (17:19431807-19431821)	Frameshift, deletion	Significant association found with body weight, adiposity and appetite	
			17:19431813, C>G	c.567G>C, p.Pro189Pro, rs851551584	Synonymous coding	No significant association found with BCS	
			17:19431820, A>AG, P187PE	c.559_560insG, p.Gln187Argfs, rs852143109	Frameshift, insertion		
			17:19431850, C>G, R178G	c.532C>G, p.Arg178Gly, rs852665700	Nonsynonymous, missense		
			17:19431861, A>G, K174R	c.521A>G, p.Lys174Arg, rs851770148	Nonsynonymous, missense		
			17:19431983, C>G	c.399C>T, p.Arg133Arg	Synonymous coding		
			17:19432208, G>A	c.174G>A, p.Thr58Thr	Synonymous coding		
			17:19432226, C>A	c.156C>A, p.Pro52Pro, rs851959389	Synonymous coding		

			17:19432391, GGGCGAGCTCCTG > -	N/A	Intronic deletion		
			17:19432427, C>G	-	Intronic		
			17:19432428, A>C	-	Intronic		
			17:19434068, T>C	-	Intronic		
MC4R	*Canis familiaris:* various	CHORI-82: Canine Boxer (F) (C. familiaris) BAC Library	G637T	c.637G>T, p.Val213Phe, rs852614811	Non-synonymous, missense	No association test conducted in dogs	Skorczyk et al., 2007 [2]
			T777C	c.777T>C, p.Ala259Ala, rs851987283	Synonymous coding		
			(+33)G	c.*33C>G, rs851539399	3'UTR		
	Canis familiaris; golden retriever	N/A	c.637G>T	c.637G>T, p.Val213Phe, rs852614811	Non-synonymous, missense	No significant association found with morphological measurements	van den Berg et al., 2010 [3]
			c.777T>C	c.777T>C, p.Ala259Ala, rs851987283	Synonymous coding	No significant association found with morphological measurements	
			c.*33C>G	c.*33C>G, rs851539399	3'UTR	No association test conducted	
			c.868C>T	c.868C>T, p.Leu290Leu, rs851062983	Synonymous coding	No significant association found with morphological measurements	
	Canis familiaris; beagle	CHORI-82: Canine Boxer (F) (C. familiaris) BAC Library	C895T	c.777T>C, p.Ala259Ala, rs851987283	Synonymous coding	Significant association found with body weight in females	Zeng et al., 2014 [4]
			A420C	c.302C>A, p.Thr101Asn	Non-synonymous, missense	Significant association found with body weight	
	Canis familiaris; Labrador	CanFam 3.1	1:16132817, G>T, S330I	c.989G>T, p.Ser330Ile	Non-synonymous, missense	No significant association found with BCS	Raffan et al., 2016 [1]

	retriever, flat coated retriever		1:16133054, C>T	c.*227C>T	3’UTR		
			1:16132465, G>T, V213F	c.637G>T, p. Val213Phe, rs852614811	Non-synonymous, missense		
			1:16132860, C>G	c.*33C>G, rs851539399	3’UTR		
			1:16132605, T>C	c.777T>C, p.Ala259Ala, rs851987283	Synonymous coding		
	Canis familiaris; Labrador retriever, golden retriever, beagle, cocker spaniel	CanFam 3.1	c.637G>T, p.Val213Phe, rs852614811	c.637G>T, p.Val213Phe, rs852614811	Non-synonymous, missense	Significant association found with BCS in beagles *only*	Mankowska et al, 2017 [5]
			c.777T>C, rs851987283	c.777T>C, p.Ala259Ala, rs851987283	Synonymous coding	Significant association found with BCS in golden retrievers *only*	
			c.868C>T, rs851062983	c.868C>T, p.Leu290Leu , rs851062983	Synonymous coding	Significant association found with BCS in golden retrievers *only*	
			c.*33C>G, rs851539399	c.*33C>G, rs851539399	3’UTR	Significant association found with BCS in golden retrievers *only*	
			c.*227C>T	c.*227C>T	3’UTR	No significant association found with BCS in any breed	
			c.-435T>C, rs852471376	c.-435T>C, rs852471376	5’flanking	Significant association found with BCS in beagles *only*	
			c.301A>C **not found in genotyped cohort but referred to	c.302C>A, p.Thr101Asn	Non-synonymous, missense	No association test conducted	

	Felis catus; domestic short haired, Burmese	Felis_catus-6.2	c.92 C>T	c.92 C>T, p.Leu31Pro, rs783632116	Non-synonymous, missense	Significant association found with obese diabetics vs nondiabetics in DSH cats only	Forcada et al, 2014 [6]
			c.297C>T	c.297C>T, p.Ser99Ser	Synonymous coding	No association test conducted	
			c.303C>T	c.303C>T, p.Thr101Thr, rs785927510	Synonymous coding	No association test conducted	
FTO	*Canis familiaris:* various	CHORI-82: Canine Boxer (F) (C. familiaris) BAC Library	23 C/T, Thr1Met	c.23C>T, p.Thr8Met, rs852870212	Non-synonymous, missense	No association test conducted in dogs	Grzes et al., 2011 [7]
			I1; 192 A/T	-	Intronic		
			I1; 223 T/C	-	Intronic		
			378053 G/A	-	3′ flanking		
			378284 T/C	-	3′ flanking		
			378318 G/C	-	3′ flanking		
MC3R	*Canis familiaris:* various	CHORI-82: Canine Boxer (F) (C. familiaris) BAC Library	c.-90T(11_13); 5′ flanking	c.-90delT, rs853092001	5′UTR, deletion	No association test conducted in dogs	Skorczyk et al., 2011 [8]
			c.142C>T	c.142C>T, p.Leu48Leu, rs8916554	Synonymous coding		
INSIG2	*Canis familiaris;* various	CHORI-82: Canine Boxer (F) (C. familiaris) BAC Library	–91 G/A; 5′ flanking	c.-90G>A, rs852813691	5′ UTR	No association test conducted in dogs	Grzes et al., 2011 [7]
			c.-1C>T; 5′ flanking	c.-1C>T	5′ UTR		
			40 C/A; Arg>Ser	c.40C>A, p.Arg14Ser, rs850773724	Non-synonymous, missense		
			I1; 1483 A/T	-	Intronic		
			I1; 1637 C/T	-	Intronic		
			I1; 2169 G/A	-	Intronic		
			I4; 10820 T/A	-	Intronic		
GPR120/FFAR4	*Canis familiaris;* various	CanFam 3.1	c.252C>G, p.Ala84Ala	c.252C>G, p.Ala84Ala, rs852631320	Synonymous coding	No association test conducted	Miyabe et al., 2015 [9]
			c.282C>G, p.Asp94Asp	c.282C>G, p.Pro94Pro, rs851850900	Synonymous coding	No association test conducted	

			c.702A>G, p.Thr234Thr	c.702A>G, p.Thr234Thr	Synonymous coding	No association test conducted	
			c.726G>A, p.Thr242Thr	c.726G>A, p.Thr242Thr	Synonymous coding	No association test conducted	
			c.984T>C, p.Asn328Asn	c.984T>C, p.Asn328Asn, rs852472019	Synonymous coding	No association test conducted	
			c.287T>G, p.Leu96Arg	c.287T>G, p.Leu96Arg	Non-synonymous, missense	No association test conducted	
			c.307G>A, p.Ala103Thr	c.307G>A, p.Ala103Thr	Non-synonymous, missense	No association test conducted	
			c.446G>C, p.Gly149Ala	c.446G>C, p.Gly149Ala	Non-synonymous, missense	No association test conducted	
			c.595C>A, p.Pro199Thr	c.595A>C, p.Thr199Pro, rs853030954	Non-synonymous, missense	Significant association found with body condition	
PPARG	*Canis familiaris*; various inc, mongrels	CanFam 2.0	E7, C1362T	N/A	N/A	No association test conducted	Nishii et al., 2007 [10]
TNF	*Canis familiaris*; various inc. mongrels	N/A	c.-40A>C	c.-40A>C, rs22216187	5′ UTR	Significant association found with body condition in Labrador retrievers *only*	Mankowska et al., 2016 [11]
			c.249C>T	c.249C>T, p.Thr83Thr, rs9095590	Synonymous coding	No association test conducted	
			c.548A>T, p.Glu183Val	c.548A>T, p.Glu183Val, rs851883595	Non-synonymous, missense	No association test conducted	
			c.627C>T	c.627C>T, p.Ser209Ser, rs850991197	Synonymous coding	No association test conducted	
			c.186+16A>G	-	Intronic	No association test conducted	
			c.186 +174GAAT(5_7)	-	Intronic, insertion	No association test conducted	

			c.186+211C>T	-	Intronic	No association test conducted
			c.187-47T>C	-	Intronic	No association test conducted
			c.233-54T>C	-	Intronic	No association test conducted
			c.233+14G>A	-	Intronic	Significant association found with body condition in Labrador retrievers *only*
			c.233+17G>T	-	Intronic	No association test conducted
			c.233+108A>G	-	Intronic	No association test conducted
			c.*107G>A	c.*107G>A, rs852784980	3′ UTR	No association test conducted
IL6	*Canis familiaris*; various inc. mongrels	N/A	c.102T>C	N/A	Synonymous coding	No association test conducted
			c.572G>A	N/A	Synonymous coding	No association test conducted
			c.309+215T>C	-	Intronic	No significant association found with BCS
			c.*283G>A	c.*283G>A, rs850980833	3′ UTR	No association test conducted
RETN	*Canis familiaris*; various inc. mongrels	N/A	c.75G>A	c.75G>A, p.Glu25Glu, rs852185407	Synonymous coding	No significant association found with BCS
			c.141C>T	c.141C>T, p.Gly47Gly, rs852329356	Synonymous coding	No association test conducted
			Synonymous coding: c.236C>G	c.236C>G, p.Ser79Cys, rs851766760	Non-synonymous, missense	No association test conducted
			c.19C>T, p.Leu7Phe	c.19C>T, p.Leu7Phe, rs852470997	Non-synonymous, missense	No association test conducted

			c.194–69T>A	c.194-69T>A, rs853182485	Intronic	No significant association found with BCS	
			c.115+29G>C;	-	Intronic	No association test conducted	
			c.115+143T>G	-	Intronic	No association test conducted	
			c.116-179G>A	-	Intronic	No association test conducted	
FAM174A	*Equus ferus caballus;* Arabian horses	EquCab2.0	BIEC2-262534, chr14:69276814 T > C	N/A	Intronic	Associated with various metabolic measures	Lewis et al. 2016/2017 [12, 13]
			FAM174A 11-G	N/A	3' UTR		

References

[1] E. Raffan *et al.*, "A Deletion in the Canine POMC Gene Is Associated with Weight and Appetite in Obesity-Prone Labrador Retriever Dogs," *Cell Metab,* vol. 23, no. 5, pp. 893-900, May 10 2016, doi: 10.1016/j.cmet.2016.04.012.

[2] A. Skorczyk *et al.*, "Polymorphism and chromosomal location of the MC4R (melanocortin-4 receptor) gene in the dog and red fox," *Gene,* vol. 392, no. 1-2, pp. 247-52, May 1 2007, doi: 10.1016/j.gene.2006.12.027.

[3] L. van den Berg *et al.*, "Analysis of variation in the melanocortin-4 receptor gene (mc4r) in Golden Retriever dogs," *Anim Genet,* vol. 41, no. 5, p. 557, Oct 2010, doi: 10.1111/j.1365-2052.2010.02049.x.

[4] R. Zeng, Y. Zhang, and P. Du, "SNPs of melanocortin 4 receptor (MC4R) associated with body weight in Beagle dogs," *Exp Anim,* vol. 63, no. 1, pp. 73-8, 2014, doi: 10.1538/expanim.63.73.

[5] M. Mankowska, J. Nowacka-Woszuk, A. Graczyk, P. Ciazynska, M. Stachowiak, and M. Switonski, "Polymorphism and methylation of the MC4R gene in obese and non-obese dogs," *Mol Biol Rep,* vol. 44, no. 4, pp. 333-339, Aug 2017, doi: 10.1007/s11033-017-4114-3.

[6] Y. Forcada, A. Holder, D. B. Church, and B. Catchpole, "A polymorphism in the melanocortin 4 receptor gene (MC4R:c.92C>T) is associated with diabetes mellitus in overweight domestic shorthaired cats," *J Vet Intern Med,* vol. 28, no. 2, pp. 458-64, Mar-Apr 2014, doi: 10.1111/jvim.12275.

[7] M. Grzes, I. Szczerbal, H. Fijak-Nowak, M. Szydlowski, and M. Switonski, "Two candidate genes (FTO and INSIG2) for fat accumulation in four canids: chromosome mapping, gene polymorphisms and association studies of body and skin weight of red foxes," *Cytogenet Genome Res,* vol. 135, no. 1, pp. 25-32, 2011, doi: 10.1159/000330457.

[8] A. Skorczyk, K. Flisikowski, M. Szydlowski, J. Cieslak, R. Fries, and M. Switonski, "Association of MC3R gene polymorphisms with body weight in the red fox and comparative gene organization in four canids," *Anim Genet,* vol. 42, no. 1, pp. 104-7, Feb 2011, doi: 10.1111/j.1365-2052.2010.02075.x.

[9] M. Miyabe *et al.*, "Genetic variants of the unsaturated fatty acid receptor GPR120 relating to obesity in dogs," *J Vet Med Sci,* vol. 77, no. 10, pp. 1201-6, Oct 2015, doi: 10.1292/jvms.15-0031.

[10] N. Nishii *et al.*, "Cloning, expression and investigation for polymorphisms of canine peroxisome proliferator-activated receptors," *Comp Biochem Physiol B Biochem Mol Biol,* vol. 147, no. 4, pp. 690-7, Aug 2007, doi: 10.1016/j.cbpb.2007.04.011.

[11] M. Mankowska *et al.*, "Sequence analysis of three canine adipokine genes revealed an association between TNF polymorphisms and obesity in Labrador dogs," *Anim Genet,* vol. 47, no. 2, pp. 245-9, Apr 2016, doi: 10.1111/age.12390.

[12] S. Lewis, H. Holl, M. T. Long, M. Mallicote, and S. Brooks, "Candidate Gene and Marker for Equine Metabolic Syndrome," *Journal of Animal Science,* vol. 94, 5, pp. 166-167, 2016.

[13] S. L. Lewis *et al.*, "Genomewide association study reveals a risk locus for equine metabolic syndrome in the Arabian horse," *J Anim Sci,* vol. 95, no. 3, pp. 1071-1079, Mar 2017, doi: 10.2527/jas.2016.1221.
